# The Effect of Low-Frequency Physiological Correction on the Reproducibility and Specificity of Resting-State fMRI Metrics: Functional Connectivity, ALFF, and ReHo

**DOI:** 10.3389/fnins.2017.00546

**Published:** 2017-10-05

**Authors:** Ali M. Golestani, Jonathan B. Kwinta, Yasha B. Khatamian, J. Jean Chen

**Affiliations:** ^1^Rotman Research Institute at Baycrest Centre, University of Toronto, Toronto, ON, Canada; ^2^Department of Medical Biophysics, Faculty of Medicine, University of Toronto, Toronto, ON, Canada

**Keywords:** resting-state fMRI, physiological noise, test-retest reproducibility, sensitivity, specificity, end-tidal CO_2_, respiratory volume, heart rate variability

## Abstract

The resting-state fMRI (rs-fMRI) signal is affected by a variety of low-frequency physiological phenomena, including variations in cardiac-rate (CRV), respiratory-volume (RVT), and end-tidal CO_2_ (PETCO_2_). While these effects have become better understood in recent years, the impact that their correction has on the quality of rs-fMRI measurements has yet to be clarified. The objective of this paper is to investigate the effect of correcting for CRV, RVT and PETCO_2_ on the rs-fMRI measurements. Nine healthy subjects underwent a test-retest rs-fMRI acquisition using repetition times (TRs) of 2 s (long-TR) and 0.323 s (short-TR), and the data were processed using eight different physiological correction strategies. Subsequently, regional homogeneity (ReHo), amplitude of low-frequency fluctuation (ALFF), and resting-state connectivity of the motor and default-mode networks are calculated for each strategy. Reproducibility is calculated using intra-class correlation and the Dice Coefficient, while the accuracy of functional-connectivity measures is assessed through network separability, sensitivity and specificity. We found that: (1) the reproducibility of the rs-fMRI measures improved significantly after correction for PETCO_2_; (2) separability of functional networks increased after PETCO_2_ correction but was not affected by RVT and CRV correction; (3) the effect of physiological correction does not depend on the data sampling-rate; (4) the effect of physiological processes and correction strategies is network-specific. Our findings highlight limitations in our understanding of rs-fMRI quality measures, and underscore the importance of using multiple quality measures to determine the optimal physiological correction strategy.

## Introduction

Resting-state fMRI is typically measured through blood oxygenation level dependent (BOLD) contrast, which indirectly measures brain function through blood oxygenation changes following neuronal activity. The typical BOLD-measurement technique is gradient-echo echo-planar-imaging (GE-EPI). However, the BOLD signal contains not only neuronal contributions, but also several physiological contributions, which can either generate BOLD-related hemodynamics or introduce artifacts through interactions with the magnetic field. For instance, respiration and heartbeat generate bulk motion as well as local movement that is most pronounced in the cerebrospinal fluid (CSF), brain stem, and in the vicinity of large blood vessels (Dagli et al., 1999). In addition, respiration causes susceptibility changes in the lungs that interfere with the static magnetic field and induce shifts in the MR image, mainly in the phase-encoding direction (Hu et al., 1995; Raj et al., 2001; Pfeuffer et al., 2002; Murphy et al., 2013)—a major concern for GE-EPI.

Typically, the rs-fMRI sampling rate is ~0.5 Hz, which is only appropriate for representing signal changes up to 0.25 Hz. This is much lower than the Nyquist sampling rate required for the fundamental cardiac and respiratory frequencies (~1 and ~0.3 Hz, respectively), raising the possibility that physiological contributions to rs-fMRI measures depend on signal sampling rate. Moreover, neuronally-relevant information in rs-fMRI data is commonly identified with the low-frequency range (below 0.1 Hz; Cordes et al., 2001), which is shared with low-frequency physiological fluctuations. The most common examples of these include cardiac rate variation (CRV), respiratory volume per unit time (RVT) and pressure of end-tidal CO_2_ fluctuations (PETCO_2_). RVT is mainly localized in the gray matter, specifically in regions with high vascular density, including the occipital region and the default mode network (DMN; Birn et al., 2006). On the other hand, the effect of CRV is strongest in brain regions close to arteries and CSF (Chang et al., 2009). Finally, fluctuations in arterial pressure of CO_2_, which can be indirectly measured through PETCO_2_, alter the BOLD signal through vasodilatory and constrictive action. Like the RVT effect, the PETCO_2_ effect is dominant in the gray matter (Wise et al., 2004; Chang and Glover, 2009; Golestani et al., 2015).

In the context of rs-fMRI, these physiological effects have generally been considered as artifacts from non-neuronal sources that can mimic BOLD signal fluctuations and connectivity, potentially reducing the reliability and neuronal-specificity of rs-fMRI measures. Some excellent works in recent years established the theoretical foundation for investigating and removing these physiological effects from the rs-fMRI signal (Birn et al., 2006; Chang et al., 2009). Specifically, the typical procedure is to record the corresponding physiological signals during the rs-fMRI data acquisition, model their effects on the BOLD signal and eliminate them using regression (Birn et al., 2006; Chang et al., 2009; Golestani et al., 2015). However, little is known about the effect of the correction on the quality of rs-fMRI measures, and indeed, the consequences of different physiological corrections.

In the rs-fMRI literature, the accuracy of rs-fMRI measures is typically assessed based on their test-retest reproducibility, commonly quantified through the intra-class correlation coefficient (ICC; Anderson et al., 2011b; Chou et al., 2012; Faria et al., 2012; Zuo and Xing, 2014). ICC is defined as the ratio of inter-subject variance to total variance (inter-subject + inter-session variance). If within-subject inter-session variance were considerably smaller than inter-subject variance, ICC would be close to one, which is inferred as high reproducibility. Previous studies of various rs-fMRI measures have shown moderate to high reproducibility, depending on the measure (Zuo and Xing, 2014). That is, measures such as amplitude of low frequency fluctuations (ALFF; Zuo et al., 2010a) and regional homogeneity (ReHo; Zuo et al., 2013) are highly reproducible across sessions, whereas connectivity metrics derived from graph-theoretical network analysis are considered not very reproducible (Wang et al., 2011). Moreover, the reproducibility of connectivity maps is sensitive to acquisition length, the number of time points included (Birn et al., 2013; Liao et al., 2013), the sampling rate (Liao et al., 2013) and of course the processing steps (Franco et al., 2013; Zuo et al., 2013). The ICC, however, only assesses the reproducibility of the connectivity values but not that of network extent. The latter has previously been assessed using the Dice Similarity Coefficient (Amemiya et al., 2014; Ganger et al., 2015; Jann et al., 2015). The Dice Coefficient compares the spatial extent of different connectivity maps, and a Dice Coefficient close to unity reflects high overlap between two maps, hence high spatial reproducibility.

Notwithstanding the current emphasis on reproducibility as the chief quality measure, high reproducibility does not equal to high measurement quality. For instance, we should also like to be able to distinguish between the areas that are part of a network from those outside of it (i.e., high sensitivity and specificity). Yet, sensitivity and specificity has been largely overlooked in the literature, as they are more difficult to assess. In that respect, while the true individual resting-state connectivity map is unknown, a number of resting-state functional networks have been consistently found in various populations (Damoiseaux et al., 2006; Yeo et al., 2011). The resulting group-based network atlases, which are arguably less affected by physiological artifacts compared to the individual subject-level maps, are presumably more robust and representative of true functional networks. Thus, we may now have a means to quantify the sensitivity and specificity of rs-fMRI connectivity maps.

To the authors' best knowledge, there exists only one prior study addressing the effect of various physiological corrections on rs-fMRI measurement quality, despite the importance of the topic (Birn et al., 2014). Interestingly, the findings suggest that physiological correction may have little or even a negative effect on the reproducibility of the fMRI connectivity patterns. To explain this surprising finding, the authors skilfully demonstrated that physiological correction reduces both within- and between-subject variance, resulting in an overall ICC reduction. Despite this observation, the authors recommend removing the physiological effects from the BOLD signal, as the physiological correction would potentially increase the validity of the rs-fMRI connectivity studies. Moreover, the authors correctly admitted in the paper that the accuracy of using a global physiological regressor for physiological correction is questionable, given the evident inter-subject and regional variability in the BOLD physiological response (Falahpour et al., 2013; Cordes et al., 2014; Golestani et al., 2015). Nevertheless, this work leaves unanswered a number of important questions. First, it only addressed the effects of CRV and RVT. Given recent evidence of the unique effects of PETCO_2_ fluctuations on rs-fMRI (Golestani et al., 2015), the effect of PETCO_2_ correction should also be addressed. Second, the study relied solely on reproducibility as a metric of merit, and used ICC as the only measure of reproducibility, neglecting other aspects of rs-fMRI data quality. In addition, the study focused on functional connectivity measurements and did not consider other commonly used rs-fMRI measures such as ReHo and ALFF.

In this paper, we investigate the effect of a number of correction strategies involving three low-frequency physiological signal sources (CRV, RVT, and PETCO_2_) on the rs-fMRI measurements. The novelties of this study are: (1) we study the effect of PETCO_2_ correction in addition to CRV and RVT correction; (2) we estimate and eliminate the effect of physiological modulations using a voxel-wise instead of a global approach, accounting for potential inter-subject and inter-regional variability; (3) in assessing reproducibility, we use not only the ICC, but also the Dice Coefficient; (4) in addition to reproducibility, we measure the sensitivity and specificity of the resting-state connectivity maps with the help of a resting-state connectivity template (Yeo et al., 2011); (5) we also assess the separability of the connectivity maps by calculating relative-connectivity of within-network connectivity to between-network connectivity; (6) we include ReHo and ALFF in addition to resting-state connectivity in our assessments; (7) we investigate the effect of fMRI acquisition sampling rate on the efficacy of physiological corrections.

## Methods

### Participants and data acquisition

Nine healthy subjects participated in this study (3 male; mean age = 26 ± 5.8 years). Participants were recruited from Baycrest and local communities through the Baycrest Participants Database. The study was approved by the research ethics board (REB) of Baycrest, and the consent obtained from all participants was both written and informed, in accordance with the Declaration of Helsinki.

All images were acquired using a Siemens TIM Trio 3 Tesla System (Siemens, Erlangen, Germany), with a 32-channel phased-array head coil for reception and body-coil transmission. We acquired rs-fMRI data using multiple repetition times (TR) to investigate the effect of sampling rate. Each TR was used in two sessions to allow assessment of test-retest reproducibility. Specifically, the “long-TR” protocol involved conventional single-shot gradient-echo echo-planar imaging (GRE-EPI; TR = 2,000 ms, TE = 30 ms, flip angle = 90°, 26 slices, 0.6 mm between-slice gap, 3.44 × 3.44 × 4.6 mm^3^ voxels, matrix size: 64 × 64 × 26, 240 frames), while the “short-TR” protocol involved slice-accelerated (Feinberg et al., 2010; Setsompop et al., 2012) single-shot GRE-EPI [TR = 323 ms, TE = 30 ms, flip angle = 40°, 15 slices, 1 mm between-slice gap, 3.44 × 3.44 × 6 mm^3^, matrix size = 64 × 64 × 15, 1,850 frames, acceleration factor = 3, phase encoding shift factor = 2, with “leak block” (Cauley et al., 2014) and a GRAPPA reconstruction kernel of 3 × 3]. Participants were instructed to close their eyes but remain awake during the functional scans. Furthermore, T1-weighted anatomical images were collected for cross-subject registration (MPRAGE, TR = 2,400 ms, TE = 2.43 ms, FOV = 256 mm, TI = 1,000 ms, readout bandwidth = 180 Hz/px, voxel size = 1 × 1 × 1 mm^3^).

### Image processing

To achieve consistency in data lengths, the initial 2 min of the short-TR data is discarded, yielding 8 min per run for both long- and short-TR datasets. Furthermore, as short-TR and long-TR data acquisitions differed in more than TR, we created a downsampled version of the short-TR data to specifically target the effect of sampling rate. This was done by temporally decimating the original short-TR data to (2,000 ms/323 ms) times the original sample rate, so as to match the sampling interval of the “long-TR” data. Resting-state fMRI processing was carried out using FMRIB software library (FSL, publicly available at www.fmrib.ox.ac.uk/fsl). The preprocessing pipeline included motion correction (Jenkinson et al., 2002), brain extraction (Smith, 2002), spatial smoothing (10 mm FWHM), frequency filtering (see section *Resting-State fMRI Measures* for details) and regression of six motion parameters. Time-locked cardiac and respiratory effects were also removed using RETROICOR (Glover et al., 2000) implemented in AFNI (AFNI: http://afni.nimh.nih.gov/afni).

### Physiological monitoring and correction

The details on measuring, modeling, and correcting for the physiological signals are explained in our previous paper (Golestani et al., 2015). In short, we accounted for the effects of the following three physiological signals:

- *Cardiac-rate variation (CRV)*: The cardiac signal was recorded using the scanner's built-in pulse oximeter, connected to the subject's index finger. CRV is defined as the time interval between consecutive R peaks, averaged in a 4-s window (Chang et al., 2009).
- *Respiration volume per unit time (RVT)*: The respiratory-depth correlated signal was recorded using an elastic belt connected to a BioPac system (BioPac, Goleta, USA), placed just below the subject's ribcage (Birn et al., 2006; Chang and Glover, 2009; Golestani et al., 2015). A piezoelectric sensor within the belt measured the extent of extension and contraction in the belt caused by exhales and inhales. It is assumed that the respiration volume is linearly related to the amplitude of the belt signal. RVT was calculated as the ratio of breathing depth (estimated from the local maxima and minima of the respiratory waveform) over a given time period (Birn et al., 2006).
- *End-tidal CO*_2_ *(PETCO*_2_*)*: CO_2_ level in the subject's breathing was measured using a BioPac system. A mask covering the mouth and nose of the subject was connected via plastic tubing to the BioPac's CO_2_ sensor. PETCO_2_ signal was computed as the breath-by-breath maxima of the CO_2_ tracing.

At each TR, these three physiological signals were re-sampled to correspond to the sampling rate of the rs-fMRI data. Subsequently, BOLD response functions to the three physiological signals were estimated for each voxel in the brain volume, as explained in our previous work (Golestani et al., 2015). In short, the voxel-wise BOLD responses to the three physiological signals were simultaneously estimated using a Gaussian model. The estimated responses were then used to correct the effect of these physiological signals. The physiological correction involved the convolution of the physiological signals with the corresponding estimated responses, the inclusion of the convolved response into a voxel-wise linear regression and regressing out a given physiological effect of interest from the BOLD signal. In total, eight different physiological correction combinations were applied:

- “Base”: no correction;
- “PETCO_2_,” “CRV,” and “RVT”: each of these three settings involved correcting for only one of the three physiological effects;
- “PETCO_2_+CRV,” “PETCO_2_+RVT,” and “CRV+RVT”: each of these three settings involved correcting for a set of two physiological effects;
- “All”: whereby all of the three physiological signals were corrected for.

We did not orthogonalize the physiological signals with respect to one another as we did in our previous work (Golestani et al., 2015), as the goal is to maximally remove noise instead of estimate their response functions.

### Resting-state fMRI measures

#### Amplitude of low-frequency fluctuation (ALFF)

ALFF is defined as the sum of amplitudes of each voxel's signal frequency spectrum within the low-frequency range (Zang et al., 2007) and reflects the amplitude of spontaneous low-frequency fluctuations in the BOLD signal. To eliminate possible effects of low-pass filtering on the rs-fMRI frequency spectrum, datasets with no temporal filtering were used to estimate ALFF. The unfiltered rs-fMRI signal is transformed into the frequency domain using the Fourier transform, and the spectrum in the frequency range of 0.01–0.1 Hz is averaged to calculate ALFF. The Resting-state fMRI Data Analysis Toolkit (REST V1.8, publicly available at http://restfmri.net; Song et al., 2011) was used to calculate the ALFF maps. To allow direct comparison of ALFF values generated using long- and short-TR data, each ALFF map was normalized (subtracting the global mean then dividing by the global standard deviation; Xi et al., 2012). This normalization eliminates biases from inter-subject ALFF variability caused by differences in imaging parameters (such as sampling-rate and flip angle) between long- and short-TR acquisitions.

#### Regional homogeneity (ReHo)

ReHo is defined as the Kendall's coefficient of concordance between a given voxel and its 27 neighboring voxels (Zang et al., 2004) and represents the synchronization between the time series of a given voxel and its neighbors. This measure was also calculated using the REST toolkit. The long-TR data is spatially resampled to the same resolution as the short-TR data prior to ReHo calculations. The rs-fMRI time series was high-pass filtered (to >0.01 Hz) and low-pass filtered (<0.1 Hz) prior to the computations.

#### Functional connectivity: motor network

The motor network was the first to be demonstrated using rs-fMRI, as found in the seminal work by Biswal et al. (1995). It can easily be validated based on anatomical landmarks, and the BOLD signal in this region has been shown more affected by respiratory modulations (Birn et al., 2008) than in many other brain regions, including the default-mode network. To simplify the delineation of the motor network, we used seed-based analysis. That is, an ROI with radius of 4 mm was generated over the left motor cortex based on documented coordinates (Van Dijk et al., 2010). The average signal from this motor seed was used to generate correlation-based motor network connectivity maps. These connectivity scores were then corrected using the mixture-model method (Woolrich et al., 2005) as implemented in FSL. The mixture model estimates the distribution of the statistics as a mixture of a null distribution (with zero mean and unity standard deviation) and an alternative distribution. Mixture modeling is typically used when some assumptions in the statistical analysis might not be valid. Specifically, conventional assumptions about the temporal autocorrelation and noise level of the BOLD signal may not be valid in short TR images, leading to inflated *z*-values. Thus, we used mixture model to overcome this problem and effectively compare long- and short-TR results.

#### Functional connectivity: default-mode network (DMN)

To investigate whether the effect of physiological correction is network-dependent, we also assessed the effect of the physiological correction on the connectivity of the DMN. The DMN is amongst the most widely studied networks in healthy controls (Raichle and Snyder, 2007; Buckner, 2012), and DMN connectivity has been found disrupted in several brain diseases (Buckner et al., 2008; Broyd et al., 2009; Anticevic et al., 2012; Whitfield-Gabrieli and Ford, 2012). Of particular interest to this study is the fact that the spatial pattern of the DMN overlaps with brain regions most affected by low-frequency physiological modulations, particularly RVT and PETCO_2_ (Birn et al., 2006; Golestani et al., 2015). Therefore, we used the DMN as a test case to study the effect of the correction for physiological modulations on rs-fMRI functional connectivity (rs-fcMRI). Again, an ROI with a 4 mm radius was generated over the posterior cingulate cortex (PCC) using well-documented coordinates (Van Dijk et al., 2010). The regional average signal from this seed was correlated with all other voxels to generate connectivity maps, as described earlier. As before, each statistical connectivity map was then corrected using FSL's mixture modeling (Woolrich et al., 2005).

### Test-retest reproducibility

The maps of all rs-fMRI measures were transformed into the MNI standard space (MNI152, Montreal Neurological Institute). For ALFF and ReHo, ICC was calculated using the maps generated from the two runs of each subject. We assessed the ICC in seven distinct brain networks as defined in the work of Yeo et al. (2011). One realization of the atlas is loosely organized into the visual, somato-motor, dorsal attention, ventral attention, limbic, frontoparietal, and default-mode networks. As this rs-fcMRI atlas was generated from 1,000 subjects based on the most consistent functional connectivity patterns observed across all subjects, it is henceforth referred to as the “1,000-brain atlas.” For rs-fMRI functional connectivity, we chose the motor and default-mode networks only. The following two indices were computed to provide complementary reproducibility quantification.

#### Intra-class correlation coefficient (ICC)

The ICC is the most common reliability index in fMRI studies (Shehzad et al., 2009; Zuo et al., 2010a,b, 2012, 2013; Anderson et al., 2011b; Wang et al., 2011; Braun et al., 2012; Chou et al., 2012; Faria et al., 2012; Guo et al., 2012; Song et al., 2012; Birn et al., 2013, 2014; Bright and Murphy, 2013; Franco et al., 2013; Liao et al., 2013; Patriat et al., 2013; Wisner et al., 2013; Zhu et al., 2014). It is given by:

(1) $$\begin{array}{l} ICC=\frac{MS_{b}-MS_{w}}{MS_{b}+\left(k-1\right)MS_{w}} \end{array}$$

where *MS*_*b*_ is the inter-subject mean-squared variability, *MS*_*w*_ is the within-subject inter-session mean-squared variability and *k* is the number of runs (*k* = 2 in our case). As the ICC is sensitive to both inter-subject and within-subject inter-session variability, changes in either of the two would alter the ICC value, which is generally categorized into five reproducibility levels: poor (0–0.2), fair (0.2–0.4), moderate (0.4–0.6), substantial (0.6–0.8), and excellent (0.8–1; Guo et al., 2012; Zuo and Xing, 2014).

#### Dice coefficient

As shown earlier, the ICC reflects the consistency of connectivity values between runs, not necessarily that of the network spatial extent, which is also an important consideration. Thus, we include the Dice Coefficient, which has been commonly used in fMRI studies to evaluate the similarity of two spatial maps (Gorgolewski et al., 2013; Wisner et al., 2013; Zhu et al., 2013; Gross and Binder, 2014). It is defined as:

(2) $$\begin{array}{l} Dice=\frac{2\times |A\cap B|}{\left|A|+|B\right|} \end{array}$$

where *A* and *B* are the two spatial maps, A∩B is the intersection of the two maps, and |*A*| is the size (i.e., the number of voxels) of map *A*. We computed the Dice Coefficient between two runs of each subject, with each connectivity map defined as being above a mixture model-corrected *z* scores of 0.5.

### Separability index

Regarding rs-fMRI functional connectivity, maps can be evaluated based on not only reproducibility, but also on separability. That is, if the rs-fMRI connectivity map were predominantly sensitive to brain function instead of global physiological processes, we would expect it to demonstrate strong distinction between within-network connectivity and global (between-network) connectivity. To embody these two attributes in a single metric, using the “1,000-brain” functional-network atlas framework, the separability index is defined as:

(3) $$\begin{array}{l} SI=\frac{WNC-BNC}{WNC+BNC} \end{array}$$

where *WNC* is the within-network connectivity (average connectivity, e.g., z-scores, inside the network of interest) and BNC is between-network connectivity (average connectivity between the network of interest and the remaining six networks). Separability indices for the motor network and DMN were calculated for each physiological-correction strategy and then averaged across the two runs of each subject.

### Sensitivity and specificity

Using the 1,000-brain connectivity atlas as the pseudo ground-truth, sensitivity and specificity of the connectivity maps for each physiological correction strategy was calculated. Each connectivity map was defined with a mixture-model corrected threshold of 0.5. Sensitivity was calculated as the ratio of the number of voxels inside the network of interest that is correctly identified (true positives) over the total number of voxels in the network (true positives + false negatives). Specificity was calculated as the ratio of the number of gray-matter voxels outside the network of interest that correctly identified as non-connected (true negatives) over the total number of gray-matter voxels outside of the network (true negatives + false positives).

### Statistical analysis

No statistical test was carried out on the ICC values, as the entire subject group would yield a single ICC value. Thus, the ICC values were simply compared among physiological correction methods and sampling rates. For functional connectivity, other measures (Dice Coefficient, separability index, sensitivity, and specificity) were compared within DMN and MN separately using two-factor within-subject ANOVA with physiological correction strategy (“Method”) and sampling-rate (TR) as factors. In case a significant effect was observed, we also assessed the observed effect by performing follow-up *t*-tests.

## Results

One of the subjects exhibited considerable head motion during one of the rs-fMRI scans, and was thus excluded from the study, yielding total group size of 8 subjects (3 male, age 26 ± 6.2 years).

### Resting-state fMRI measures

**Figure 1:**
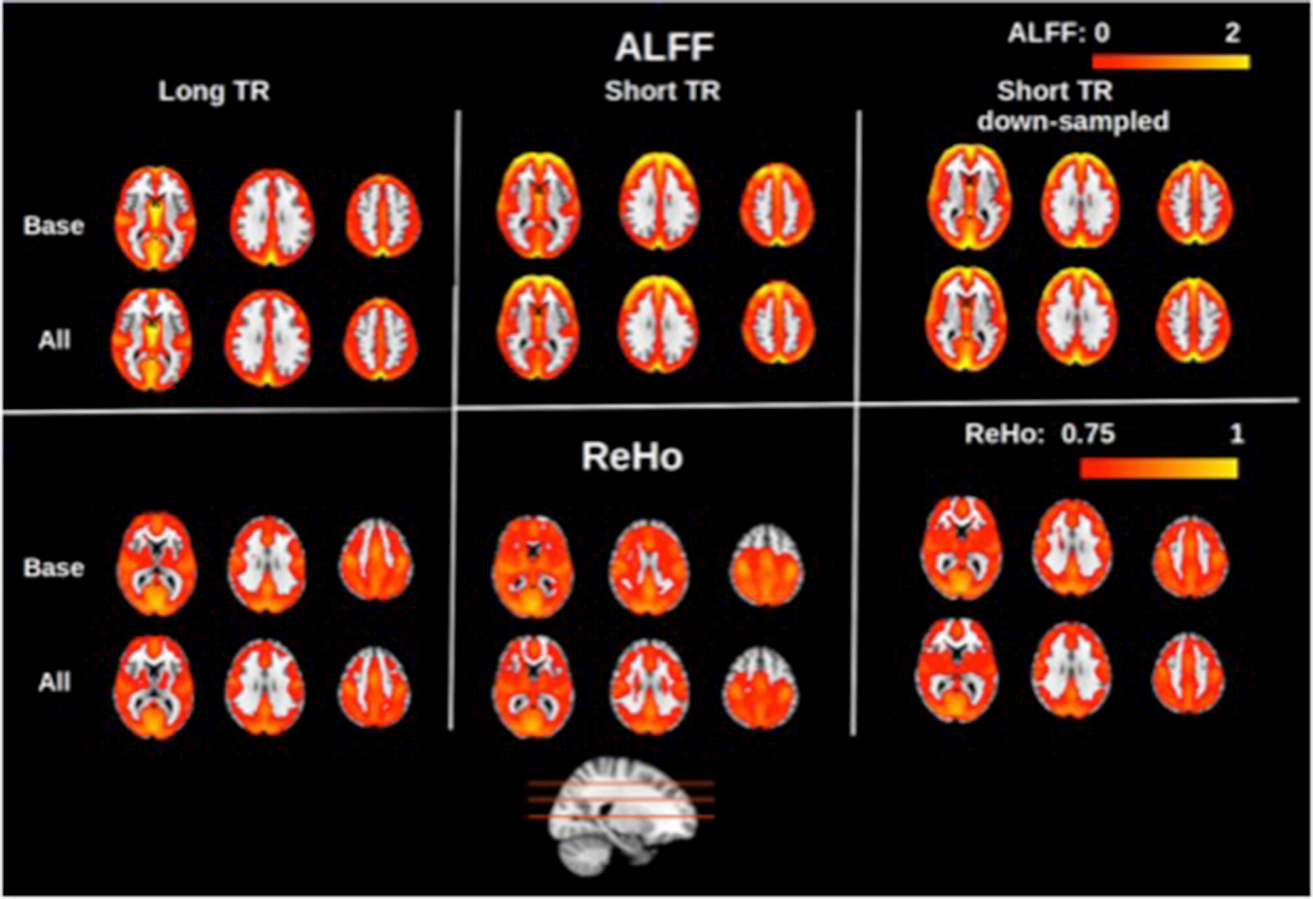
Group-averaged maps of normalized amplitude of low-frequency fluctuations (ALFF) and regional homogeneity (ReHo). Physiological correction does not have noticeable effect on the ALFF and ReHo maps, as shown by the small difference between maps generated with no physiological correction (“Base”) and with all three physiological corrections (“All”). This was the case for long-TR (TR = 2 s), short-TR (TR = 0.323 s), and down-sampled short-TR data. Gray matter is associated with considerably higher ALFF and ReHo values, specifically in the default-mode network as well as frontal and occipital regions. Sampling-rate (TR) does not considerably alters the ALFF and ReHo maps.

**Figure 2:**
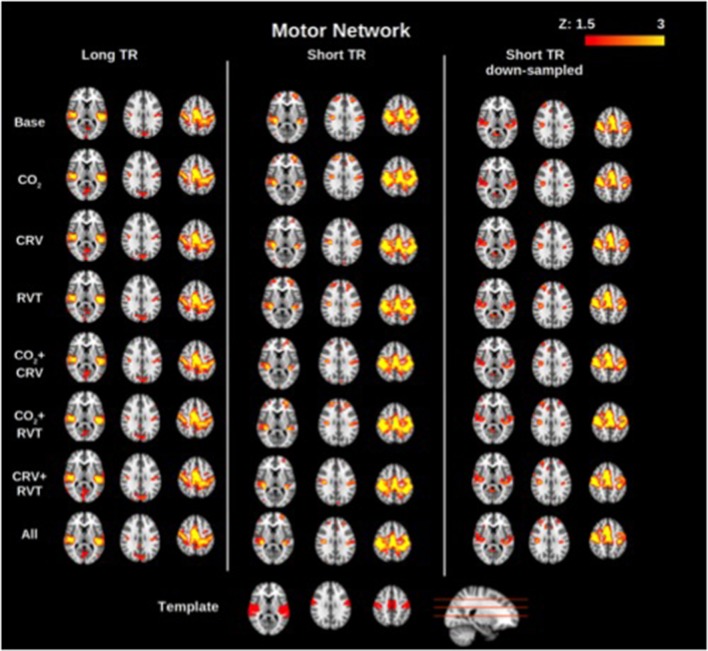
Group-averaged motor network (MN) connectivity maps generated with different physiological correction strategies, using long-TR, short-TR, and down-sampled short-TR data. A motor network template from the atlas generated by Yeo et al. (2011) is shown at the bottom for reference. CRV correction alters connectivity maps more than correction for PETCO_2_ (labeled as CO_2_) and RVT by reducing the extent of connected clusters outside the motor cortex. Sampling-rate does not seem to have a considerable effect on the connectivity maps.

**Figure 3:**
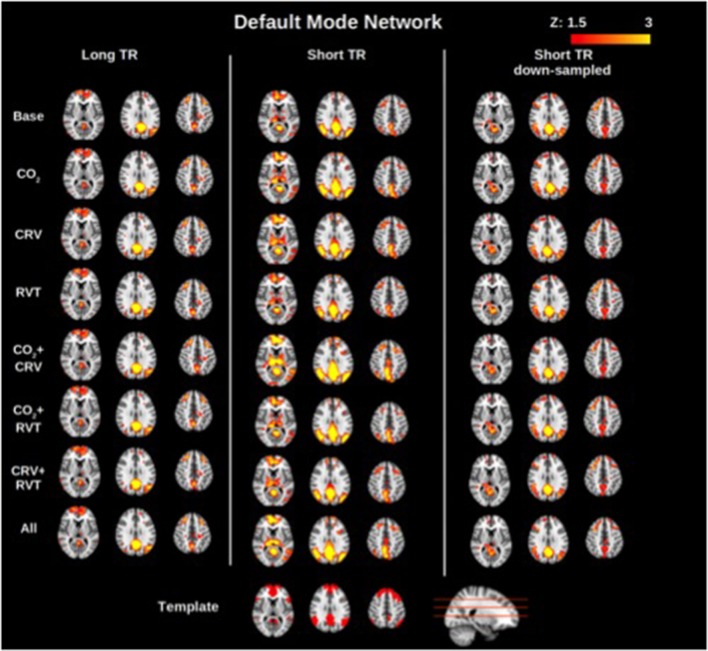
Group-averaged default mode network (DMN) connectivity maps generated with different physiological correction strategies, using long-TR, short-TR, and down-sampled short-TR data. A default mode network template from the atlas generated by Yeo et al. (2011) is shown at the bottom for reference. Physiological correction does not noticeably alter connectivity maps. As in the motor network case, connectivity maps generated from data with different sampling rates are comparable.

Group-averaged maps (averaged across all subjects and both sessions) of the rs-fMRI measures are shown in Figures 1–**3**. In Figure 1, group-averaged normalized ALFF and ReHo are shown for long-TR, short-TR, and short-TR-down-sampled datasets. Consistent with previous studies (Zang et al., 2007; Zou et al., 2008; Zuo et al., 2010a), ALFF values are higher in the gray matter, specifically in the DMN as well as occipital and frontal regions. Likewise, ReHo is considerably stronger in the gray matter than in the white matter and CSF, compatible with the previous study by Long et al. (2008). For the purposes of rs-fMRI, we found ALFF and ReHo maps to be insensitive to the choice of physiological correction method, as ALFF and ReHo maps for different physiological corrections are nearly identical. To demonstrate this point, we contrast the maps derived using no physiological correction (“Base”) with those resulting from correcting for all three physiological signals (“All”). The only noticeable effect of physiological correction is a reduction in white-matter ReHo.

Group-average motor network and DMN connectivity maps corresponding to different physiological correction strategies are shown in Figures 2, 3, respectively. Correcting for PETCO_2_ and RVT do not appear to have a considerable effect on the connectivity maps. In contrast, for both networks, the involvement of CRV correction was found to have a stronger effect, substantially reducing the size of the connected clusters, specifically outside the network of interest. The effect of CRV correction on rs-connectivity is more evident in short-TR images. Regardless of the physiological correction method, functional networks are consistently revealed, indicating that our physiological corrections do not significantly compromise the functional information in the BOLD signal. Lastly, sampling-rate does not have considerable effect on the connectivity maps.

### Test-retest reproducibility

#### ALFF

**Figure 4:**
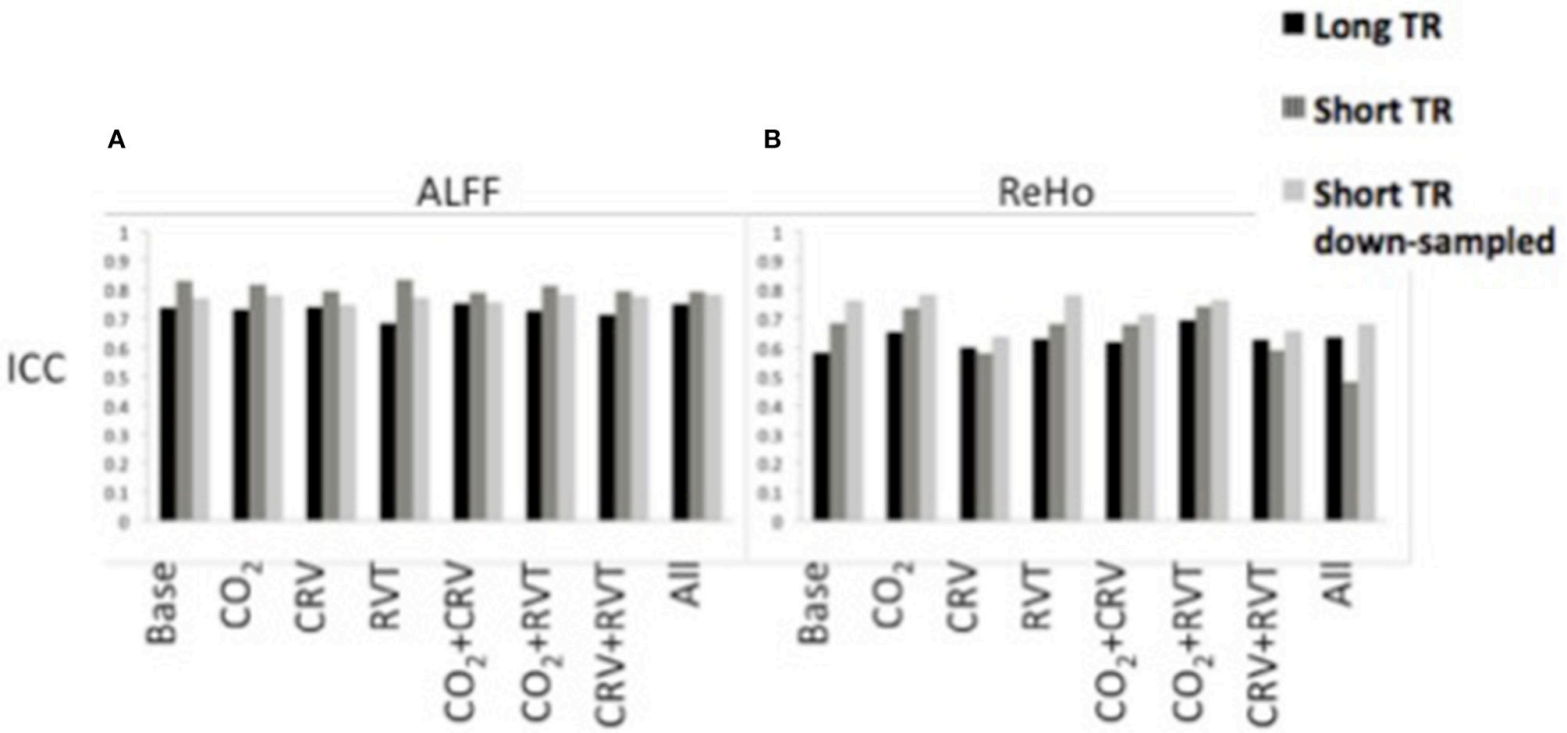
Reproducibility measures (ICC) for the normalized amplitude of low-frequency fluctuations (ALFF) **(A)** and regional homogeneity (ReHo) **(B)** for different physiological correction strategies, based on long-TR (black), short-TR (dark gray), and down-sampled short-TR (light gray) data. Both ALFF and ReHo values are highly reproducible. Physiological correction does not have a consistent effect on the reproducibility of ALFF values. PETCO_2_ (labeled as CO_2_) and RVT correction increased, and CRV correction decreased the ICC values for ReHo. Short-TR data is associated with higher reproducibility for ALFF.

ICC values associated with the ALFF are shown in Figure 4A, generated from long-TR, short-TR, and down-sampled short-TR datasets. Results show ALFF values to be highly reproducible in all cases, with ICC values consistently in the range of 0.65–0.85. For the most part, physiological correction does not considerably alter the reproducibility of ALFF values. Nonetheless, ICC values are higher for the short-TR data. However, the fact that ICC values for down-sampled short-TR data is also higher than the long-TR, shows that the sampling-rate is not the main reason for the higher ALFF reproducibility associated with short-TR data.

#### ReHo

ICC values associated with ReHo are shown in Figure 4B. The ICC values (0.5–0.8) demonstrate relatively high reproducibility across different physiological correction methods and sampling-rates. Nonetheless, different trends were observed, specifically in that PETCO_2_ and RVT correction increases the ICC whereas CRV correction decreases the ICC. Short-TR down-sampled data are consistently associated with the highest ICC values.

#### Functional connectivity: reproducibility

**Figure 5:**
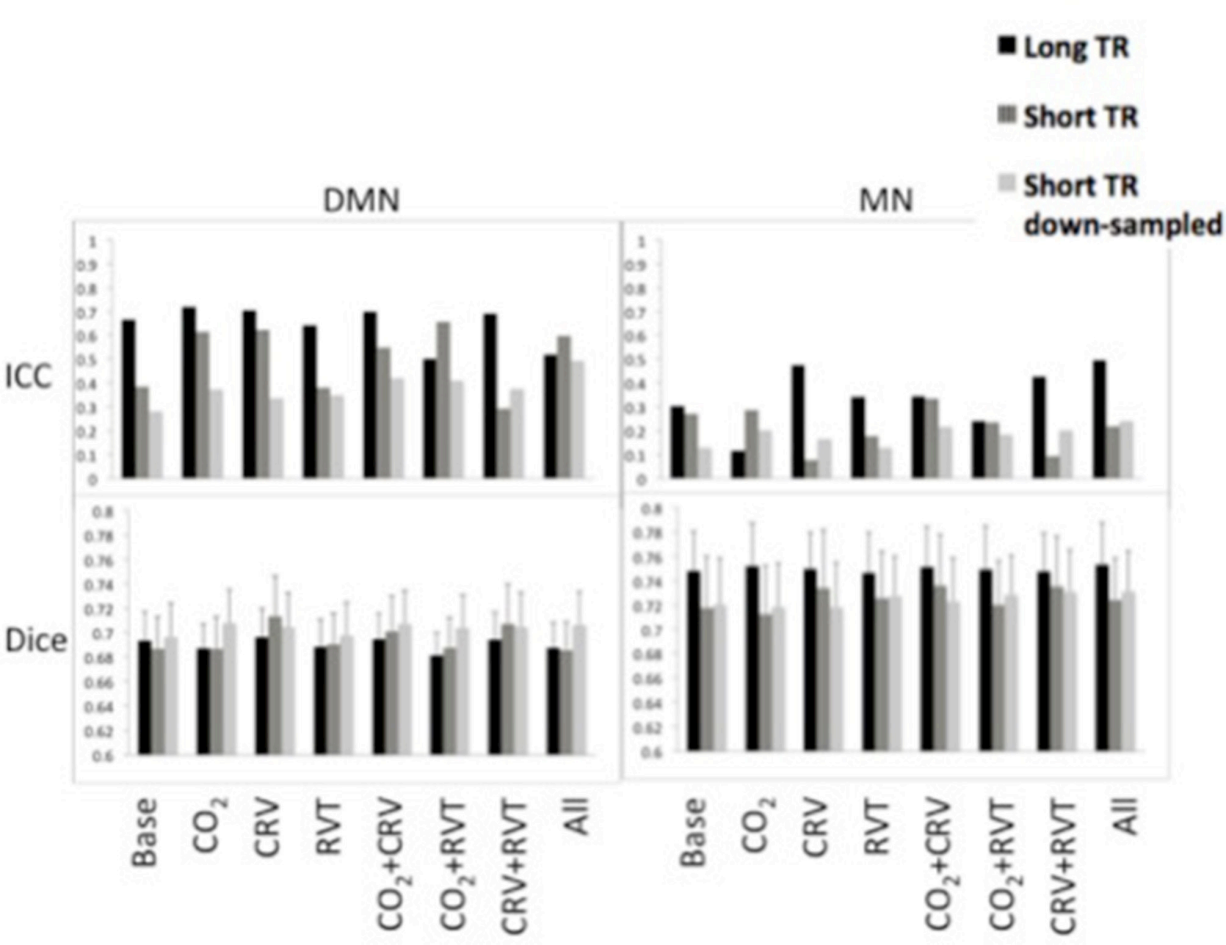
Reproducibility measures [ICC represents reproducibility of values (top) and Dice coefficient represents reproducibility of spatial maps (bottom)] for the default-mode network (DMN) (left) and motor network (MN) (right). DMN had higher reproducibility of the connectivity values in lower reproducibility of the connectivity maps compared to MN. PETCO_2_ and CRV correction increased ICC values in DMN, whereas RVT correction had no considerable effect. Physiological correction did not have a consistent effect on the ICC values in the MN. No significant difference was observed between physiological correction strategies for the reproducibility of the spatial maps. In MN, reproducibility values tended to be higher in the long-TR case. This pattern was not extensible to the DMN reproducibility.

Reproducibility measures for motor network (MN) and default-mode network (DMN) connectivity are shown in Figure 5. ICC values are between 0.25 and 0.75 for the DMN, which is substantially higher than the ICC values for the MN (0.10–0.50). Further, short-TR data is associated with lower ICC values, specifically in the DMN, suggesting that data with lower sampling-rates tend to generate connectivity values with higher reproducibility. Broadly speaking, physiological correction does not appear to alter the reproducibility of the connectivity values, except in the DMN, where correction for PETCO_2_ and CRV increase the ICC.

Spatial reproducibility of the connectivity maps is shown as Dice Coefficient plots in Figure 5. The Dice Coefficient is relatively high (0.7–0.8) showing that the DMN and MN are highly reproducible spatially. The spatial pattern of the MN is slightly more reproducible than that of the DMN. However, statistical analysis (Table 1) demonstrated no difference between physiological correction strategies, as well as different sampling-rates.

**Table 1:** Results of the statistical analysis on between-run Dice coefficient of the connectivity maps for DMN and MN.

**Factor**	**DMN**		**MN**	
	***F***	***p***	***F***	***p***
TR	*F*_(2, 14)_ = 0.23	0.802	*F*_(2, 14)_ = 0.34	0.718
Method	*F*_(7, 49)_ = 1.44	0.211	*F*_(7, 49)_ = 0.78	0.601

*Two-factor, within-subject ANOVA test showed no significant effect of sampling rate (TR) or physiological correction strategy (Method)*.

#### Separability index

**Figure 6:**
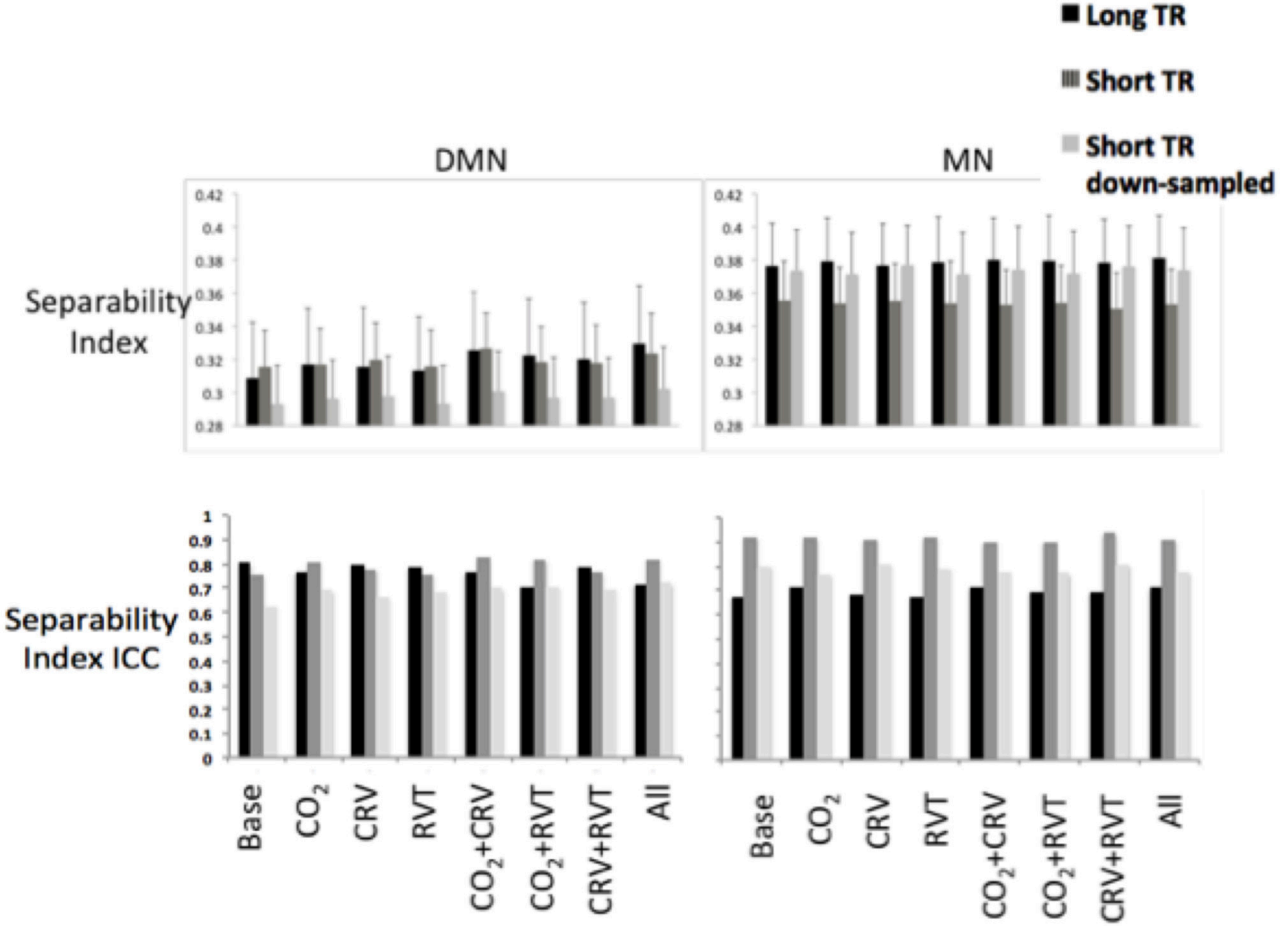
Separability index values for default-mode network (DMN) (left) and motor network (MN) (right) as a result of various physiological correction strategies. Separability index was higher in the MN compared to the DMN. Physiological corrections had a small effect on the separability index in the MN. PETCO_2_ correction increased the separability index in the DMN. Sampling rate did not significantly change the separability index in neither DMN nor MN. The ICC of the separability on the other hand, was considerably higher for separability indices generated from short-TR images.

Separability indices are summarized in Figure 6 for the MN and the DMN, generated from long-TR, short-TR, and down-sampled short-TR datasets. The MN exhibited significantly higher separability indices than the DMN, for both long- and short-TR cases (0.37 ± 0.043 for the MN vs. 0.31 ± 0.038 for the DMN, *p* = 0.037). The ANOVA (Table 2) showed that the sampling-rate does not significantly influence the separability index in the two networks. With respect to the choice of physiological-correction method, two-way ANOVA within the MN does not reveal any significant effects, but this is not the case for the DMN. Follow-up *t*-tests reveal that correcting for PETCO_2_ significantly increases the separability index of the DMN. As indicated by high ICC values (ICC > 0.6), the separability indices are highly reproducible. Also, higher sampling rate (short TR) is associated with higher reproducibility of the separability indices, specifically in the MN.

**Table 2:** Results of the statistical analysis on the separability index for DMN and MN.

**(A)**							
	**DMN**				**MN**		
**Factor**	***F***			***p***	***F***		***p***
TR	*F*_(2, 14)_ = 0.36			0.706	*F*_(2, 14)_ = 0.99		0.398
Method	*F*_(7, 49)_ = 4.02			<0.001	*F*_(7, 49)_ = 0.07		0.999
**(B)**							
**Method**	**CO**_2_	**CRV**	**RVT**	**CO**_2_**CRV**	**CO**_2_**RVT**	**CRVRVT**	**All**
Base	0.011	0.149	0.245	0.012	0.015	0.113	0.043
CO_2_		0.778	0.275	0.071	0.337	0.661	0.165
CRV			0.324	0.001	0.635	0.753	0.109
RVT				0.025	0.025	0.175	0.052
CO_2_CRV					0.117	0.014	0.824
CO_2_RVT						0.667	0.118
CRVRVT							0.042

*(A) Two-factor, within-subject ANOVA test showed the physiological correction method had significant effect on the separability index in the DMN. (B) Follow-up t-test revealed that PETCO_2_ correction significantly increased the separability index in the DMN (green shows the cases in which the method in the column gives significantly higher values compared to the method in the row, red shows the cases in which the method in the column gives significantly smaller values compared to the method in the row)*.

#### Sensitivity and specificity

**Figure 7:**
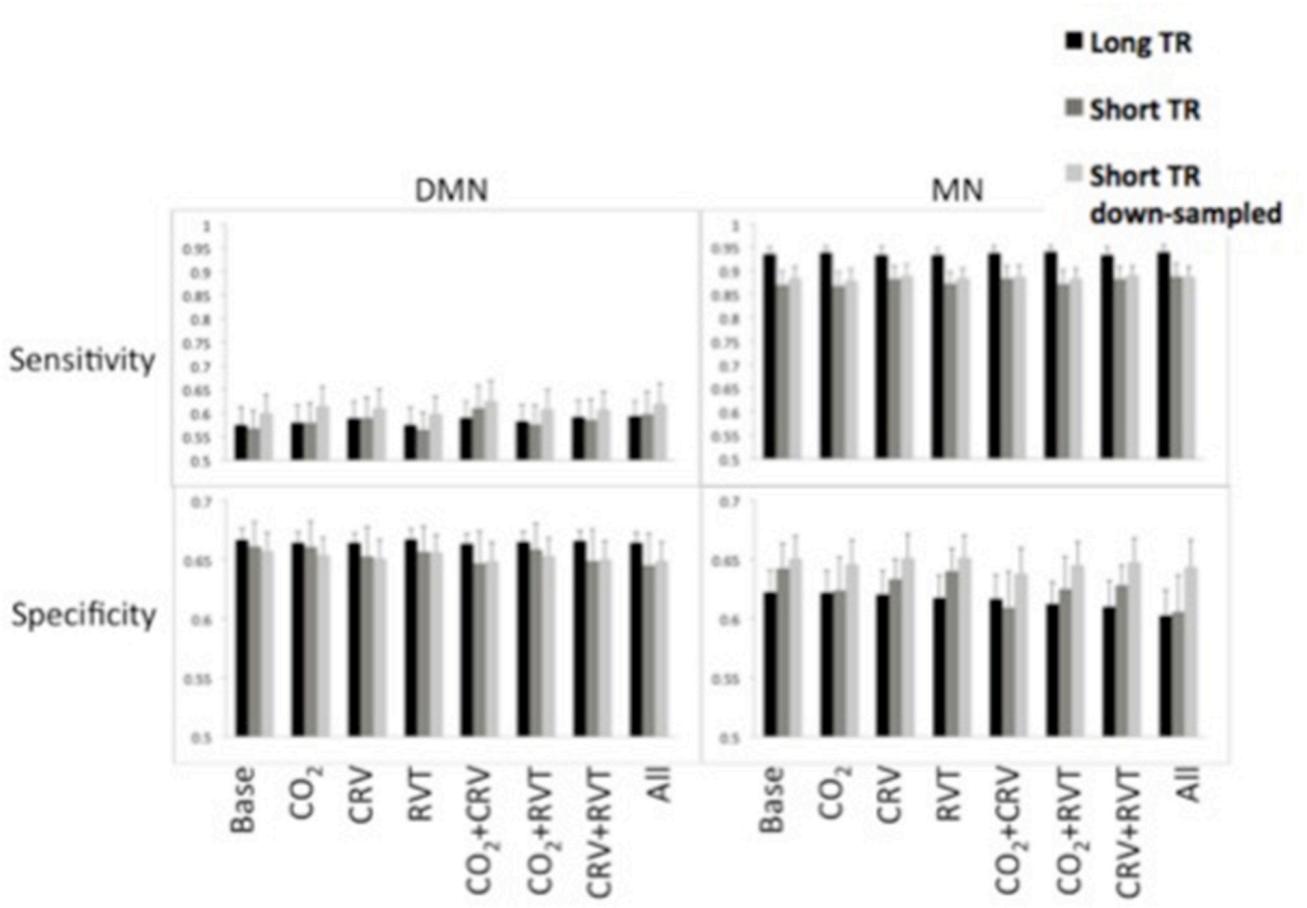
Sensitivity (top) and specificity (bottom) measures of the default-mode network (DMN) (left) and motor network (MN) (right) for different physiological correction strategies, based on long-TR (black), short-TR (dark gray), and down-sampled short-TR (light gray) data. DMN demonstrated lower sensitivity and higher specificity compared to MN. CRV correction increased sensitivity and decrease specificity of DMN connectivity maps. Physiological correction did not considerably alter the sensitivity of MN connectivity maps and tended to reduce their specificity. For the MN, long-TR data was associated with higher sensitivity and lower specificity.

Sensitivity and specificity of the DMN and MN are shown in Figure 7. The MN is associated with significantly higher detection sensitivity than the DMN (0.89 ± 0.054 for MN vs. 0.59 ± 0.082 for DMN, *p* < 0.001). Specificity of the DMN is higher than for the MN, although the difference is not significant (0.66 ± 0.035 for DMN and 0.63 ± 0.047 for MN, *p* = 0.23).

In Table 3 we summarize the statistical test results showing that the MN maps generated from long-TR data are associated with significantly higher sensitivity than those generated from short-TR or down-sampled short-TR data. In contrast, sampling-rate does not affect sensitivity or specificity of mapping the DMN. With respect to the choice of physiological correction method, CRV correction significantly improves the sensitivity of the DMN connectivity maps, but the sensitivity of the MN is not significantly associated with any form of physiological correction.

**Table 3:** Results of the statistical analysis on the sensitivity of DMN and MN connectivity maps.

**(A)**							
	**DMN**				**MN**		
**Factor**	***F***			***p***	***F***		***p***
TR	*F*_(2, 14)_ = 0.22			0.802	*F*_(2, 14)_ = 5.38		0.018
Method	*F*_(7, 49)_ = 6.13			<0.001	*F*_(7, 49)_ = 1.77		0.114
**(B)**							
**Method**	**CO**_2_	**CRV**	**RVT**	**CO**_2_**CRV**	**CO**_2_**RVT**	**CRVRVT**	**All**
Base	0.785	0.010	0.505	0.009	0.212	0.040	0.050
CO_2_		0.434	0.045	0.023	0.181	0.606	0.081
CRV			0.006	0.042	0.272	0.509	0.381
RVT				0.005	0.104	0.013	0.027
CO_2_CRV					0.015	0.010	0.315
CO_2_RVT						0.384	0.037
CRVRVT							0.216
**(C)**							
**TR**				**Short TR**			**Resampled**
Long TR				0.038			0.048
Short TR							0.519

*(A) Two-factor, within-subject ANOVA test showed the physiological correction method had significant effect on the sensitivity of the DMN. In addition, sampling rate had significant effect on the sensitivity of the MN. (B) Follow-up t-test on the effect of physiological correction method showed that CRV correction significantly increased the sensitivity of DMN connectivity maps, (C) t-tests on the effect of the sampling-rate demonstrated that long-TR data generated MN connectivity maps with higher sensitivity compared to short-TR and down-sampled short-TR data. (Green shows the cases in which the method in the column gives significantly higher values compared to the method in the row, red shows the cases in which the method in the column gives significantly smaller values compared to the method in the row)*.

Statistical results on the specificity of the connectivity maps are shown in Table 4. Sampling-rate does not affect on the specificity of the DMN and MN connectivity maps. However, CRV correction tends to reduce the specificity of the connectivity maps in both the DMN and MN, although the effect is not consistently significant.

**Table 4:** Results of the statistical analysis on the specificity of the DMN and MN connectivity maps.

**(A)**							
	**DMN**				**MN**		
**Factor**	***F***			***p***	***F***		***p***
TR	*F*_(2, 14)_ = 0.24			0.794	*F*_(2, 14)_ = 1.13		0.349
Method	*F*_(7, 49)_ = 2.61			0.022	*F*_(7, 49)_ = 2.99		0.011
**(B)**							
**Method**	**CO**_2_	**CRV**	**RVT**	**CO**_2_**CRV**	**CO**_2_**RVT**	**CRVRVT**	**All**
Base	0.431	0.088	0.490	0.090	0.359	0.153	0.099
CO_2_		0.248	0.886	0.090	0.666	0.238	0.135
CRV			0.157	0.279	0.346	0.633	0.351
RVT				0.059	0.462	0.096	0.040
CO_2_CRV					0.095	0.349	0.902
CO_2_RVT						0.240	0.078
CRVRVT							0.251
**(C)**							
**Method**	**CO**_2_	**CRV**	**RVT**	**CO**_2_**CRV**	**CO**_2_**RVT**	**CRVRVT**	**All**
Base	0.182	0.268	0.447	0.068	0.126	0.037	0.048
CO_2_		0.537	0.351	0.024	0.251	0.741	0.022
CRV			0.727	0.152	0.341	0.244	0.110
RVT				0.106	0.165	0.027	0.057
CO_2_CRV					0.096	0.405	0.274
CO_2_RVT						0.858	0.011
CRVRVT							0.193

*(A) Two-factor, within-subject ANOVA test showed the physiological correction method had significant effect on the specificity of DMN and MN. (B) Follow-up t-test on the effect of physiological correction on the DMN specificity. Although not significant, CRV correction tends to deteriorate the DMN specificity. The only observed significant difference was between correction for all three physiological signals and correction for RVT, where correction for all of the physiological signals decreased the specificity of the DMN. (C) Follow-up t-test on the effect of physiological correction on the MN specificity demonstrated that in general, correction for the CRV effect decreased the specificity of the MN. (Red shows the cases in which the method in the column gives significantly smaller values compared to the method in the row)*.

## Discussion

Low-frequency physiological effects can contribute significantly to the BOLD fMRI signal and undermine the accuracy and reliability of fMRI measures, especially in the resting state. However, as mentioned earlier, despite considerable research devoted to studying the reliability of resting-state fMRI measures (Shehzad et al., 2009; Zuo et al., 2010a,b, 2012, 2013; Anderson et al., 2011b; Wang et al., 2011; Braun et al., 2012; Chou et al., 2012; Faria et al., 2012; Guo et al., 2012; Song et al., 2012; Birn et al., 2013; Bright and Murphy, 2013; Franco et al., 2013; Liao et al., 2013; Patriat et al., 2013; Wisner et al., 2013; Zhu et al., 2014), studies that investigate the effect of physiological correction on the accuracy and reproducibility of rs-fMRI measures are extremely limited.

In this study, we determine the effect of various low-frequency physiological correction strategies on the reproducibility, sensitivity and specificity of rs-fMRI measures. The main findings are: (1) PETCO_2_ correction has the most consistent positive effect on the reproducibility of rs-fMRI metrics; (2) PETCO_2_ correction has the most significant positive effect on the separability of functional connectivity maps; (3) the effect of physiological correction is not influenced by fMRI data sampling rate; (4) there is substantial variability between different brain regions and networks in terms of the impact of physiological correction. Specifically: (1) Physiological correction has a stronger effect on the DMN compare to the MN; (2) CRV correction increases the reproducibility but decreases the specificity of the DMN connectivity maps; moreover, it decreases the reproducibility of the ReHo values. These findings are summarized in Table 5. Our findings highlight limitations in our understanding of rs-fMRI quality measures, and underscore the importance of using multiple quality measures to determine the optimal physiological correction strategy. In particular, we argue against the simplification of rs-fMRI data quality based on reproducibility alone. We discuss these findings in detail as follows.

**Table 5:** Summary of the effects of different physiological corrections on rs-fMRI measures: (A) Reproducibility of ALFF and ReHo, (B) rs-connectivity measures in the default mode network, (C) rs-connectivity measures in the motor network.

**(A)**					
**ICC**	**ALFF**				**ReHo**
CO_2_	↔				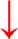
CRV	↔				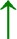
RVT	↔				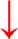
**(B)**					
**DMN**	**ICC**	**Dice**	**Separability**	**Sensitivity**	**Specificity**
CO_2_	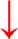	↔	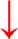	↔	↔
CRV	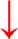	↔	↔	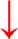	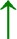
RVT	↔	↔	↔	↔	↔
**(C)**					
**MN**	**ICC**	**Dice**	**Separability**	**Sensitivity**	**Specificity**
CO_2_	↔	↔	↔	↔	↔
CRV	↔	↔	↔	↔	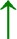
RVT	↔	↔	↔	↔	↔

*(Green arrow shows the cases in which the rs-fMRI measure is increased due to the correction method, red arrow shows cases in which the rs-fMRI measure is decreased due to the correction method)*.

### Data analysis quality without physiological correction

#### Reproducibility

We found the reproducibility of rs-fMRI measures to be highly dependent on the type of rs-fMRI measure in question. The ALFF shows substantial reproducibility in the gray matter independent of physiological correction method and sampling rate, supported by other studies (Zuo et al., 2010a; Li et al., 2012). As the ALFF measures the power of low-frequency BOLD signal fluctuations, which presumably reflects the magnitude of neural activity (Yang et al., 2007; Zou et al., 2008; Yan and Zang, 2010), we expect to observe higher ALFF values and reproducibility in the gray matter. Likewise, as expected, ReHo values are higher in the gray matter compared to in the white matter and CSF, as ReHo is a measure of local homogeneity in brain activity, which is most meaningfully measured in the gray matter (Li et al., 2012). ReHo is also the most reproducible when based on the down-sampled short-TR data, judging from the ICC values. In fact, this could be related to the fact that the down-sampled data was associated with fewer time points and hence higher ReHo values.

Unlike for ALFF and ReHo, the reproducibility indices of which were measured in all of the seven functional networks, the reproducibility of rs-fMRI functional connectivity was considered within two networks of interest, namely the motor and default-mode networks, which differ vastly in terms of their cytoarchitectonic and functional traits. These networks were chosen to present a snapshot of the network-dependence of functional connectivity measures in our investigation. The moderate ICC values echo findings from previous studies (Braun et al., 2012; Franco et al., 2013; Wisner et al., 2013). Our finding of higher ICC values for the DMN compared to the MN also supports previous findings (Shehzad et al., 2009; Zuo et al., 2010b). Moreover, reproducibility of the connectivity maps measured by the Dice Coefficient is higher for the MN than for the DMN, in agreement with previous studies (Zhu et al., 2013). This suggests that although overall the connectivity values in the DMN are relatively stable, the spatial pattern changes is not as stable. Indeed, it has been reported that DMN connectivity map is more sensitive to the level of vigilance and to uncontrolled brain activations (Kucyi and Davis, 2014; Zalesky et al., 2014). As a case in point, it has been shown that (Demertzi et al., 2011) hypnosis increases connectivity between middle frontal and angular gyri and decreases connectivity between posterior and parahippocampal structures, which are encompassed in the DMN. Moreover, sleep deprivation may cause disconnection between posterior cingulate and other nodes of the DMN (Wang et al., 2015). MN connectivity maps on the other hand are not known to be affected by factors of this nature.

#### Network separability

The motor network is more spatially separable than the DMN, as represented in Figures 2, 3. This point is supported by our quantitative comparison of the separability indices between the motor network and the DMN (Figure 6). This may be due to the simpler nature of the motor network, which makes it a good test case for methodological development. Thus, the motor-network results allowed us to establish the effect of physiological correction on the spatial pattern of rs-fcMRI measurements. Nonetheless, with regards to more complex networks such as the DMN, the interpretation of separability index is less straightforward, and higher separability may not be related to higher accuracy. In fact, there exists episodic connectivity between the DMN and other networks (Smith et al., 2012; Bray et al., 2015), which may increase the correlation-based, overall global connectivity with the DMN. In such cases, the interpretation of functional connectivity measurements themselves becomes less well-defined, prompting us to refer to findings in the motor network for methodological clarifications. The separability index is highly reproducible in both the MN and DMN (Figure 6). This could be due to the normalization factor in the definition of the separability index. That is, relative connectivity is less sensitive to the parameters that might vary between different data acquisition sessions, including signal-to-noise ration (SNR) and contrast-to-noise ration (CNR; Golestani and Goodyear, 2011).

#### Sensitivity and specificity

The motor network (MN) is associated with high detection sensitivity but only moderate specificity (moderate false positives). In comparison, the detection sensitivity of the DMN is considerably lower (more false negatives). DMN connectivity maps in Figure 3 also confirm presence of false negatives in the DMN connectivity maps. This finding mirrors the DMN's low separability index and is consistent with more variable nature of the DMN (Damoiseaux et al., 2006; Kucyi and Davis, 2014; Zalesky et al., 2014). As mentioned before, the spatial pattern of the DMN maps is dynamic, and some nodes of the DMN might lose their connection to the network sporadically (Demertzi et al., 2011; Kucyi and Davis, 2014; Zalesky et al., 2014; Wang et al., 2015). In such cases the disconnected nodes would represent as false-negatives, resulting in reduced sensitivity.

### The effect of PETCO_2_ correction

Notwithstanding inter-subject and regional differences, up to 15% of the resting-state BOLD signal is explained by PETCO_2_ variations (Golestani et al., 2015). While this is a sizeable contribution, we do not expect that correcting for PETCO_2_ fluctuations would dramatically change the fMRI signal. Indeed, ALFF and ReHo (Figure 1) as well as connectivity maps (Figures 2, 3) show that PETCO_2_ correction does not qualitatively alter the spatial pattern associated with these metrics. On other hand, the fact that connectivity maps can be consistently generated using data corrected for PETCO_2_ demonstrates that correction for PETCO_2_ does not jeopardize the bulk of the neuronal information contained in the BOLD signal.

Quantitatively, we found PETCO_2_ correction to slightly improve the quality of the rs-fMRI measures, although in a manner that depends on the metric and the network in question. Specifically, PETCO_2_ correction distinctly improved the reproducibility of ReHo and DMN functional connectivity values, as well as improving the separability of the DMN. This could be taken as evidence for the successful suppression of reproducible but spurious correlation between non-connected brain regions. On the other hand, the sensitivity and specificity of the resting-state connectivity maps was relatively independent of PETCO_2_ correction (Figure 7, Tables 3, 4). We only considered the gray-matter regions in our sensitivity and specificity calculations, as the PETCO_2_ effect on the BOLD signal is dominant in the gray matter (Wise et al., 2004; Chang and Glover, 2009; Golestani et al., 2015). Our finding indicates that PETCO_2_ correction may have a more global affect that does not distinguish between networks.

We note that in this study, we assume that resting-state PETCO_2_ fluctuations are independent of neuronal activity. Indeed, elimination of the PETCO_2_ effect did not change resting-state connectivity maps in any major way, but such an assumption may not always hold. In fact, previous studies have shown that the level of arousal is associated with both neural activity level and PETCO_2_ level (Dahan and Teppema, 2003; Kotajima et al., 2005). Even a subtle difference in the resting state, such as between eyes-open and eyes-closed states, can alter the vascular reactivity to PETCO_2_ (Peng et al., 2013). Nevertheless, the fact that PETCO_2_ correction does not considerably alter the rs-fMRI maps shows the possible interaction between PETCO_2_ signal and brain activation is not considerable, at least in our experiment. On the other hand, while the improvements in reproducibility brought about by PETCO_2_ correction are fairly consistent, one must bear in mind that reproducibility may not always be the best aim, given the natural neural variabilities that were discussed earlier.

### Effects of CRV and RVT correction

CRV correction appears to improve the quality of resting-state connectivity maps, specifically in the DMN. Many of the brain regions affected by CRV located in the realm of the DMN (Chang et al., 2009; Golestani et al., 2015), e.g., the PCC, medial frontal cortex, and the angular gyrus. The effect of CRV correction on the resting-state connectivity is more pronounced for the short-TR images. Other studies (Faraji-Dana et al., accepted) have also reported stronger CRV contribution to multiband EPI data, as compared to conventional EPI data, the mechanism however is not clear. The reproducibility of the ReHo decreased after CRV correction. On the other hand, the reproducibility of DMN connectivity improved after CRV correction, which is apparently contrary to previous findings by Birn et al. (2014). The most likely explanation for this discrepancy is the fact that we performed voxel-wise estimation of the CRV and RVT response and correction for its effect, whereas Birn et al. (2014) used either no convolution with a response function or in some cases a single global response function averaged across several subjects. As shown in a number of recent works (Falahpour et al., 2013; Cordes et al., 2014; Golestani et al., 2015), inter-subject and inter-regional variance in the CRV and RVT response function is significant, and should be accounted for in physiological corrections. Accurately estimating and eliminating CRV effect removes a source of signal modulation irrelevant to brain connectivity and generates more reproducible connectivity values.

Despite its positive effect on the reproducibility of DMN connectivity values, CRV correction reduces the specificity of the DMN connectivity maps (Figure 7, Table 4). A potential reason is that the CRV effects may have been over-corrected in regions exhibiting lower CRV dependence, resulting in additional artificial correlations. Therefore, we posit that although correcting for CRV improves measurement reproducibility, it can lead to lower specificity, potentially compromising the accuracy of DMN maps. On the other hand, the 1,000-brain atlas, which served as reference, was generated without CRV correction, and therefore potentially contains CRV-related biases. Nonetheless, CRV correction does not significantly affect motor network connectivity.

In the case of RVT correction, no consistent or significant effect on the rs-fMRI reproducibility is found in our study. This is also in contrast to what has been reported by Birn et al. (2014), whereby RVT correction decreased ICC values. Apart from potential between-subject and between-region differences in the RVT responses that explained before, differences in the resting-state paradigm may also be the cause of this discrepancy. We used eyes-closed resting-state, whereas Birn et al. (2014) used an eyes-open resting-state paradigm. Indeed, recent studies have demonstrated that the RVT signal and hence its effect on the BOLD signal differ between eyes-open and eyes-closed conditions (Yuan et al., 2013). On the other hand, RVT correction does not have a considerable impact on the separability, sensitivity, or specificity of connectivity maps. This is likely due to the rather global nature of RVT effects on the BOLD signal. RVT correction likely eliminates a synchronously oscillating part of the BOLD signal not only from voxels inside DMN but also from elsewhere in the gray matter. Consequently, RVT correction reduces both within- and between-network correlations.

### The effect of rs-fMRI sampling rate

We also targeted the effect of sampling rate using long- and short-TR data. To achieve higher temporal signal to noise ration (tSNR), we used lower flip angle (FA) for short-TR data. A lower FA however not only increases tSNR, but also reduces the effect of physiological noise on the BOLD signal (Gonzalez-Castillo et al., 2011). Moreover, to achieve whole-brain coverage with short-TR acquisitions, we used slightly thicker slice thickness, which might change the through-plane smoothness of images. Furthermore, SMS has also been known to introduce “leakage effects” that may introduce false correlations across slices (Todd et al., 2016), which may bias our findings and are unrelated to sampling rate *per-se*, as we have shown in our previous work (Faraji-Dana et al., accepted). Thus, to determine if any observed difference between the results from short- and long-TR is due to sampling rate and not to other imaging parameters, we created downsampled short-TR data and investigated the effect of sampling rate by comparing the results between the short-TR and downsampled short-TR data.

While RETROICOR correction, which targets the time-locked high-frequency noise components, has demonstrated little effect on the ICC values of functional connectivity measures (Birn et al., 2014), we hypothesized that the effect of correction for time-locked respiration and cardiac effects may be different for long- and for short-TR data. That is, the fundamental frequency peaks of the time-locked effects are captured in the short-TR data and can be directly filtered out, whereas in the long-TR data the effects alias into lower frequencies and become irremovable. Moreover, RETROICOR does not completely remove the time-locked physiological effects even in the absence of aliasing (Golestani et al., 2015). Therefore, it is plausible that long-TR data are more affected by time-locked cardiac and respiratory signals than short-TR data. Alternatively, one may argue that the short-TR data contains more time points, resulting in statistically stronger rs-fMRI maps and potentially higher reproducibility. To our surprise, the effect of sampling rate on rs-fMRI measures and the effectiveness of physiological correction was not as strong as hypothesized. A higher sampling-rate however, appears to improve the reproducibility of some fMRI measures. More specifically, images with higher sampling-rate have more reproducible separability index, specifically in the motor network. Moreover, consistent with the previous study by Zuo et al. (2013), ReHo maps generated from short-TR data is substantially more reproducible than those generated from long-TR data.

Interestingly, the sensitivity of the MN connectivity maps generated from long-TR data is significantly higher than that associated with short-TR data (Table 3). However, this cannot be directly attributed to sampling-rate, as the sensitivity of the MN connectivity map generated from down-sampled short-TR data is comparable to that of the maps generated from short-TR data (Figure 7). Therefore, other imaging parameter differences might have contributed to the observed phenomenon. For instance, the short-TR data (and by extension in the down-sampled short-TR data) were acquired using a lower flip angle, which is likely to have reduced the fMRI signal to noise ratio (SNR; Gonzalez-Castillo et al., 2011), reducing BOLD signal sensitivity.

We note that existing reproducibility studies assume that the true resting-state connectivity should be stable within subjects and therefore reproducible, whereas noise and artifacts should be more random in nature and hence their elimination would improve reproducibility. However, recent studies have shown that resting-state connectivity is dynamic and variable with time (Chang and Glover, 2010; Schaefer et al., 2014). Moreover, as physiological components in the fMRI data are in fact associated with moderate within-session ICC (Zuo et al., 2010b; Birn et al., 2014), their elimination from the fMRI signal may reduce both intra- and inter-subject variance and hence will affect the ICC in an unpredictable manner. For instance, an ICC reduction could be interpret as either increased within-subject variance or decreased between-subject variance (Birn et al., 2014). Moreover, the ICC is known to be sensitive to the data range (Muller and Buttner, 1994; Lee et al., 2012), and a larger dynamic range is associated with a higher ICC value. This is in fact a limitation of the general practice of using ICC alone to assess reproducibility, and supports our argument that higher reproducibility of rs-fMRI measures does not necessarily translate to higher rs-fMRI measurement accuracy.

In our analyses, we excluded one participant with excessive head motion. The fMRI images from the remaining participants underwent typical motion correction steps (affine motion correction and regression of 6 motion parameters). Recent studies have shown that even a small head motion can create spurious local correlation in resting-state fMRI data (Power et al., 2012). Even-though we did not explicitly correct for such minute motion, we believe our findings are not influenced by head motion or the choice of motion-correction strategy, as the study design uses each data set as its own reference. That is, we assess the impact of physiological corrections only, and do not compare across data sets that may have had different motion contributions or motion correction.

### Limitations

We recognize a number of limitations of this study, many of which are limitations in the field in general.

In the effort to better characterize fcMRI data quality, we additionally measured the sensitivity, specificity and separability of the connectivity maps using the 1,000-brain functional-connectivity atlas as pseudo-ground-truth. In doing so, however, we assumed negligible between-subject variability in the spatial pattern of the rs-fMRI connectivity maps. Moreover, we assumed that physiological effects that are more global in nature do not closely reflect neuronal signaling. However, this assumption may only be appropriate in specific networks, such as those related to lower-level brain function (Anderson et al., 2011a; Manoliu et al., 2013), such as the motor network. Another concern is that when estimating network spatial extent, the necessary z-score thresholding may have affected the outcome (Bennett and Miller, 2010), particularly for the Dice Coefficient, sensitivity, and specificity measures. Here too, we hope that by interpreting our findings based on multiple quality-assessment metrics, we are providing a more complete and less biased picture. To further this line of research, we feel that experimental designs that involve alternative measures of neuronal communication are the most promising avenue.

Under the heading “*The Effect of PETCO*_*2*_,” we discussed possible interaction between the neuronal activity and PETCO_2_ fluctuations. Similar interactions may apply to CRV and RVT, as suggested by a number of previous studies (Shea, 1996; Birn et al., 2009; Macefield, 2009). While these studies and our own have recommended the removal of global physiological signals to improve the reliability of rs-fMRI measures, the relationship between these signals and rs-fMRI signal is still actively investigated.

Moreover, in this study, we used eyes-closed resting-state paradigm. Resting-state connectivity is shown to be more reliable during eyes-open condition (Patriat et al., 2013). Further studies are required to investigate if physiological correction would have different effects on eyes-open vs. eyes-closed fMRI data. As we were not able to gauge the participants' wakefulness, we are unable to comment on the effect of the vigilance variability in our findings. Notwithstanding, investigating the influence of resting-state condition and arousal level on the physiological artifact correction is part of our future work.

While we used a relatively small sample size (*N* = 8), such sizes are not uncommon amongst fMRI reproducibility studies. For instance, relevant previous studies have used sample sizes of 8 (Chou et al., 2012), 10 (Caceres et al., 2009), 18 (Meindl et al., 2010), 20 (Faria et al., 2012), 22 (Li et al., 2012), and 25 (Birn et al., 2014), respectively, for assessing reproducibility.

Finally, in this work we only investigated functional connectivity within the motor network and the DMN. We chose the DMN because it is strongly affected by physiological signals, specifically by RVT (Birn et al., 2006), and we chose the motor network in part due to its robustness and simplicity (Biswal et al., 1995; Yousry et al., 1995). As stated earlier, these two networks have been better studied and arguably better understood than most of the others in our 7-network template, and our choice is meant to provide a snapshot of the network-dependence in our measures. However, we recognize that further work is required to thoroughly investigate the effect of physiological correction on resting-state networks in general. This goal would require a better understand of the neuronal significance of the physiological processes.

## Conclusion

In this paper, we investigated the influence of correction for three low-frequency physiological modulations (i.e., PETCO_2_, CRV, RVT) on resting-state fMRI measurements, namely the amplitude of low-frequency fluctuations (ALFF), regional homogeneity (ReHo), and functional connectivity. To that end, we assessed metrics of test-retest reliability, network separability, measurement sensitivity, and specificity. We found that the effect of physiological correction on rs-fMRI measures is network-dependent. First, PETCO_2_ correction improved reproducibility and separability of DMN connectivity, with negligible effect on the motor network. Secondly, CRV correction improved the reproducibility but reduced the specificity of DMN connectivity maps. Overall, the motor networks appears to be less sensitive to the choice of physiological correction that the DMN. Based on these general findings, we conclude that the interaction between the rs-fMRI signal and physiological signals is complex and not easily demonstrated. Furthermore, to evaluate the extent of improvement resulting from physiological measures, multiple and complementary metrics should be employed. While further research is necessary to clarify the mechanisms of interactions between BOLD and physiological signals, we suggest correcting for the physiological effects in rs-fMRI studies when possible.

## Author contributions

AG and JC: Designed the study. AG, JK, and YK: Collected the data. AG: Analyzed data. AG and JC: Interpreted the data. AG and JC: Drafted the article.

### Conflict of interest statement

The authors declare that the research was conducted in the absence of any commercial or financial relationships that could be construed as a potential conflict of interest.
